# Acceptance Factors and Barriers to the Implementation of a Digital Intervention With Older Adults With Dementia or Caregivers: Protocol for an Umbrella Review

**DOI:** 10.2196/56584

**Published:** 2025-02-24

**Authors:** Ricardo Madeira, Dulce Esteves, Nuno Pinto, Alessandro Vercelli, Maria Vaz Patto

**Affiliations:** 1 Department of Sport Sciences University of Beira Interior Covilhã Portugal; 2 Research Center in Sports Sciences, Health Sciences and Human Development Covilhã Portugal; 3 RISE Health - Faculty of Health Sciences University of Beira Interior Covilhã Portugal; 4 University of Beira Interior Systematic Reviews Group University of Beira Interior Covilhã Portugal; 5 Faculty of Health Sciences University of Beira Interior Covilhã Portugal; 6 Department of Neuroscience Rita Levi Montalcini Neuroscience Institute Cavalieri Ottolenghi Torino Italy

**Keywords:** dementia, aging, telemedicine, implementation, digital intervention, older people, elderly, geriatrics, mobile applications, barriers, adherence, caregivers, self-management, acceptability

## Abstract

**Background:**

The increase in average life expectancy, aging, and the rise in the number of people living with dementia contribute to growing interest from the scientific community. As the disease progresses, people with dementia may need help with most daily activities and need to be supervised by their carer to ensure their safety. With the help of technology, health care provides new means of self-managing health that support active aging, allowing older people and people with dementia to live independently in their homes for a longer period of time. Although some systematic reviews have revealed some of the impacts of using digital interventions in this area, a broad systematic review that examines the overall results of the effect of this intervention type is mandatory.

**Objective:**

The aim of this review is to further investigate and understand the acceptability and barriers to using technology to monitor and manage health conditions of people living with dementia and their caregivers.

**Methods:**

A review of systematic reviews on acceptability factors and barriers for people with dementia and caregivers was carried out. Interventions that assessed acceptability factors and barriers to the use of technology by people with dementia or their carers were included. Each potentially relevant systematic review was assessed in full text by a member of a team of external experts.

**Results:**

The analysis of the results will be presented in the form of a detailed table of the characteristics of the reviews included. It will also describe the technologies used and factors of acceptability and barriers to their use. The search and preliminary analysis were carried out between May 5, 2023, and August 1, 2024.

**Conclusions:**

This review will play an important role as a comprehensive, evidence-based summary of the barriers and facilitators to the use of digital interventions. This review may help to establish effective policy and clinical guideline recommendations.

## Introduction

The increase in average life expectancy and aging population give rise to numerous health problems, including a decline in physical performance; changes in sensory, cognitive, and psychological abilities; and changes in social interactions [1]. Decreased physical and cognitive function is associated with a high risk of falls, increased memory loss, and difficulty completing daily tasks such as eating, dressing, medication management, and shopping [1]. Deterioration in cognition may be important, resulting in interference with occupational, domestic, or social functioning [2], making it difficult to communicate and manage activities of daily living [3,4].

The number of people with dementia worldwide is expected to increase from 46.8 million today to approximately 131.5 million by 2050 [5-7]. According to the Global Dementia Observatory and World Health Organization (WHO), dementia is the seventh leading cause of death worldwide [8]. The impact of dementia on society is well known, so early detection, timely intervention, and prevention are extremely important [9]. This issue is attracting increasing interest from the scientific community [10] and WHO, and many policies [11] are being developed worldwide.

With advances in medicine and the aging population, there is a growing number of older adults with cognitive, motor, and sensory limitations who need support to continue to be at home and maintain some degree of autonomy. For these individuals, the alternative is to live in a residential structure for older adults, where (in theory) they will have more specialized support, free from the problem of managing a home or daily activities. However, the type of care provided is usually depersonalized, often not directed toward the disease and the patient, and often too basic and not specific to individual needs. Growing economic difficulties also imply greater difficulties in this solution. Therefore, many people choose to stay at home, being cared for by other people—family members or friends—who become informal caregivers [12].

It is increasingly important to realize how we can promote the independent living and safety of people with dementia. As the disease progresses, people with dementia may need help with most daily activities, and there is a need for caregiver supervision to ensure their safety [13,14]. However, in the face of rising demand and scarce resources, the ability of health care systems and caregivers to provide equitable, responsive, and timely postdiagnostic support on a sustainable basis is a growing concern [15].

The physical, psychological, social, and economic impacts on not only people with dementia but also their caregivers, families, and society in general are another point of alarm [12,16-18]. The characteristics and complexities of dementia give rise to concerns such as frustration, risk of psychological stress, depression, reduced quality of life (QoL), and poor health on the part of the caregiver, leaving them with a great deal of responsibility and less free time to look after themselves [13,16,17,19-21]. Individuals with dementia and their carers have a range of educational and support needs that vary throughout the different stages of dementia. Information resources, caregiver training or skills development, support groups, counseling, respite care, care coordination programs, transportation services, grocery and meal delivery, personal care, and care planning are essential to help and support caregivers and patients [14].

The COVID-19 pandemic highlighted the relevance of innovative technologies in guiding the interventions of professionals and carers and supporting frail older people. It seems essential to use technologies as a means of supporting and analyzing individual behaviors, identifying the need for and barriers to specific interventions by caregivers, and maintaining the QoL of people living with dementia and their caregivers and supporting active aging for people with dementia.

The use of the internet and digital technologies is increasing rapidly around the world, including among older adults [7,22]. Digital technology encompasses a range of interconnected innovations, including the Internet of Things with next-generation telecommunication networks (eg, 5G); big data analytics; artificial intelligence using deep learning; and blockchain technology [23]. These technologies play a crucial role in active aging, as digital interventions [10] and monitoring systems [11] have been reported to be important for enabling older people and individuals with dementia to live autonomously in their own homes for a longer period of time [10]. The complexity of behavior and context in which the most diverse situations occur makes measuring this effect challenging [11]. However, the importance of 24-hour monitoring of older people has been emphasized [11]. Technology is increasingly being used in health care, such as the provision of services, home monitoring, interactive communication (eg, between patient and doctor), the transfer of health information, and peer support [24]. The benefits of telehealth or information and communication technologies to support family carers at home are increasingly being studied [7]. In this way, digital interventions can offer several advantages, including the promotion of autonomy and self-management of health and allowing patients to maintain a certain degree of independence [12-14]. These types of digital tools can provide advantages and disadvantages of residential care by providing quick access to medical information and remote support for health care professionals, as well as reducing the need for in-person visits to doctors’ offices and hospitals, resulting in transportation and other associated costs [12-14]. However, these interventions may also present limitations, such as technical barriers due to a lack of technical knowledge among older adults and caregivers, concerns about the privacy and security of personal and medical data, and accessibility and adaptation challenges, as all older adults neither have access to nor are adapted to the use of digital technologies, especially individuals with severe sensory or cognitive disabilities [12-14].

Considering the complexity of this issue, it is extremely important to understand what problems occur at this stage of life and how the use of technology can help people with dementia maintain their independent living and QoL, improve their health, and prevent possible emergencies [11]. However, the use of this type of technology to assist and monitor people with dementia and their carers should not be accepted uncritically. Older people are often referred to as reluctant users or opponents of technological change [10], and the use of technology by carers reportedly increases their burden [15,16].

This knowledge is crucial for developing more effective and personalized strategies that can improve QoL for patients and their caregivers [13,17,19,25]. In this way, by better understanding the specific challenges faced by this population, both formal and informal caregivers can adapt technological interventions to ensure greater adherence and effectiveness, thereby promoting more integrated and patient-centered care [6,7,9,13,22].

With this umbrella review, we set out to gather the data available in the literature in detail, which will allow research teams and policymakers to identify the technologies that have been successful and the objections to their use in supporting active aging and carers. The main aim of this review is to investigate, understand, and summarize the acceptability and barriers to using technology to monitor and manage health conditions in people with dementia and their carers, using more recent data. Another aim is to contribute to a theoretical debate and offer suggestions for future research in this area of work, as well as categorize the barriers and motivations for using technology. This scientific evidence will help with clinical guideline development and support political orientations.

To the best of our knowledge, this is the first general review that specifically addresses issues related to acceptability factors and barriers to the use of digital technology in people with dementia and their caregivers. We will be able to summarize the effectiveness of digital technology to support people with dementia and their carers, which is a challenge that requires an umbrella review approach.

Several research questions will be addressed: (1) What are the barriers to implementing digital interventions for people with dementia and/or caregivers? (2) What are the facilitators in implementing digital interventions for people with dementia or caregivers? (3) What digital interventions have been proposed for people with dementia or caregivers? (4) How effective were these digital interventions in alleviating the targeted problems?

## Methods

### Study Design

With the increase in the number of publications and reviews on the use of technology by older people and people with dementia in recent years, we aimed to use an umbrella review to examine whether the evidence for our research question is consistent or contradictory. Umbrella reviews focus on research questions or clinical practice for which there is a rich, high-quality evidence base. By reviewing all the existing literature on our research questions, we will be able to provide an overview of the main research findings.

To implement this umbrella review, we used the Preferred Reporting Items for Overviews of Review (PRIOR) statement protocol [26]. We selected and analyzed the studies according to the PRISMA (Preferred Reporting Items for Systematic Reviews and Meta-Analyses) guidelines. We used the Cochrane PICOS framework (population, intervention, comparison, outcomes, and study design; Table 1). The methods used in all stages of screening, selection and extraction, quality assessment, overlap management, analysis, and synthesis were referenced to ensure that our analysis can be replicated.

**Table 1:** Eligibility criteria for the inclusion of systematic reviews and meta-analyses.

Category			Specific criteria
**Inclusion criteria**			
	Publication type, date, and language	Systematic reviews (or meta-analyses) published in a peer-reviewed journal	
	Study design	Systematic reviews and meta-analyses	
	Population	Male or female individuals with dementia (age ≥60 years) or their caregivers	
	Intervention	Interventions based on the evaluation of the acceptability and barriers to implement digital interventions for people with dementia or their caregivers	
	Outcomes	Outcomes will be extracted from systematic reviews that include these aspects. Reviews including effectiveness of implementing digital interventions for people with dementia or their caregivers (eg, Results from the Unified Theory of Acceptance and Use of Technology Questionnaire, Telehealth Satisfaction Questionnaire) and among them, if available, strategies impacting the implementation process (eg, results from the Mini-Mental State Examination, Zarit Burden Interview, Impact of Caregiving Scale, Caregiver Strain Instrument)	
**Exclusion criteria**			
	Study design	Articles that were not systematic reviews and meta-analyses were not included.	
	Time period	Reviews that used studies carried out during the COVID-19 pandemic	
	Intervention	Reviews using articles with robot intervention	

### Target Population

We included systematic reviews with or without a meta-analysis focusing on acceptability factors and barriers to the use of technology by people with dementia or carers. The inclusion and exclusion criteria for this umbrella review are presented in Table 1.

Our population includes adults aged 60 years and older with dementia who had been included in studies using technologies. We did not limit the place (eg, home, clinics, academic centers, nursing home, rural populations) of intervention or the type of intervention carried out. We excluded analyses measuring acceptability factors and barriers of studies performed during the COVID-19 pandemic. The reason for this exclusion was to reduce the influence of compulsion or technology being the only way to access the intervention, which could influence the wanted results.

### Intervention, Comparators, and Outcomes

We included all systematic literature reviews that present results on the factors that can limit or enhance the use of technology by older people with dementia and carers. Among these, we focused on factors of acceptability and barriers to use and implementation using technologies. This umbrella review focuses on dementia interventions that are delivered using digital technology. For the purpose of this review, digital technology is defined as technology interventions that use smartphone apps, wearable technology, or mobile text messaging to deliver health care. These digital technologies are developed to support the independence of older people living in the community, alleviating or preventing functional or cognitive impairment, thus limiting the impact of dementia on older people and their caregivers.

The following comparisons were analyzed: technology versus control (no technology), intervention environments, type of technology used, and intervention by carers. Where possible, the intervention of caregivers will be divided into formal and informal caregivers, including factors of acceptability and barriers to the use of technology. The outcomes will be divided into outcomes of people with dementia and outcomes of caregivers. Outcomes of people with dementia include the sociodemographic characteristics of the patients, cost efficiency of using the intervention, changes in health outcomes after using the intervention (eg, Mini-Mental State Examination), patients’ adherence to and engagement with the intervention, adverse events, and barriers to using the intervention (eg, The Unified Theory of Acceptance and Use of Technology Questionnaire, Telehealth Satisfaction Questionnaire). Outcomes of caregivers include the quality and reliability of the interventions (eg, Zarit Burden Interview, Impact of Caregiving Scale, Caregiver Strain Instrument), patients’ follow-up with health care services after using the intervention, caregivers' adherence to and engagement with the intervention, adverse events, and barriers to using the intervention (eg, Telehealth Satisfaction Questionnaire).

### Publication Type, Date, and Language

Reviews published in peer-reviewed journals were included, without any date limitations.

### Data Sources and Search Strategy

The search for this umbrella review aimed to identify all systematic reviews of the literature on our research question. ISI Web of Science, Scopus, and PubMed databases were used to search for scientific articles for the development of this umbrella review. In this search, articles were identified using keywords and MeSH words (for PubMed search), individually and/or in combination. The research strategy used in PubMed included the following: (((((Elderly) OR (“Older population”)) OR (“Older people”)) OR (“Geriatrics”[Mesh])) OR (“Aged”[Mesh])) OR (“Aging”[Mesh])(((“Telemedicine”[Mesh]) OR (“Mobile Applications”[Mesh])) OR (“health informatics”)) OR (“healthcare technology”)((((implementation) OR (Barriers)) OR (Acceptance)) OR (Adherence)) OR (Restriction)((((“Dementia”[Mesh]) OR (“Cognition Disorders”[Mesh])) OR (“mental deterioration*”)) OR (“cognitive impairment*”)) OR (“mild cognitive impairment*”)((((((((Elderly) OR (“Older population”)) OR (“Older people”)) OR (“Geriatrics”[Mesh])) OR (“Aged”[Mesh])) OR (“Aging”[Mesh])) AND ((((“Telemedicine”[Mesh]) OR (“Mobile Applications”[Mesh])) OR (“health informatics”)) OR (“healthcare technology”))) AND (((((implementation) OR (Barriers)) OR (Acceptance)) OR (Adherence)) OR (Restriction))) AND (((((“Dementia”[Mesh]) OR (“Cognition Disorders”[Mesh])) OR (“mental deterioration*”)) OR (“cognitive impairment*”)) OR (“mild cognitive impairment*”)).

The research strategy used in Web of Science and Scopus included ((((((((Elderly) OR (“Older population”)) OR (“Older people”)) OR (“Geriatrics”)) OR (“Aged”)) OR (“Aging”)) AND ((((“Telemedicine”) OR (“Mobile Applications”)) OR (“health informatics”)) OR (“healthcare technology”))) AND (((((implementation) OR (Barriers)) OR (Acceptance)) OR (Adherence)) OR (Restriction))) AND (((((“Dementia”) OR (“Cognition Disorders”)) OR (“mental deterioration*”)) OR (“cognitive impairment*”)) OR (“mild cognitive impairment*”)).

### Data Exclusion

Data from the included studies were analyzed by 2 independent reviewers (MVP and RM) and extracted into Microsoft Excel according to the acceptability and barriers of new technologies, reported by people with dementia or their caregivers. Discrepancies will be resolved through discussion with a third author (NP).

### Data Synthesis

Results analysis will aim to present the technology used and the factors of acceptability and barriers to its use to support people with mild cognitive impairment or dementia and their carers. It will describe in detail each of the studies analyzed, presenting the study population, site of the intervention, time of the intervention, technology used and its category, evaluation instruments, and site. It will also describe the intervention that was carried out, reasons for acceptability and barriers, impact of the intervention, and limitations or future research with people with dementia and their carers. If possible, we will use the Consolidated Framework for Implementation Research (CFIR) to organize and analyze the factors of acceptance, barriers, and impact of support technologies for people with mild cognitive impairment or dementia and their caregivers. The CFIR tool provides a comprehensive theoretical framework that will help categorize and interpret the results, promoting a deeper understanding of the factors that influence the acceptance of and challenges with implementing technologies for people with mild cognitive impairment and dementia as well as their caregivers.

### Methodological Quality and Risk of Bias Assessment

The methodological quality of all the included studies was assessed using the AMSTAR-2 (A Measurement Tool to Assess Systematic Reviews) checklist [27]. The tool helps categorize the quality of reviews according to 7 critical areas and 9 noncritical areas. The assessment will be grouped into low, moderate, and high critical quality categories.

### Patient and Public Involvement

No patients nor members of the public were involved in the development of this comprehensive review. However, the scope and methods of this review were based on the literature and discussed with experts.

### Ethical Considerations and Dissemination

The proposed comprehensive review was based exclusively on published data from secondary sources and therefore did not involve any interactions with human beings.

The results of the umbrella review will be presented at international conferences in the fields of, for example, gerontology and geriatrics, health informatics, primary care, public health, and social sciences and will be published in a journal aimed at a wide audience. When the results are published, we will make the data generated by our research openly and publicly available. The team also intends to use social networks and the institutional websites of its research centers to disseminate its findings through websites, social media, and newsletters.

## Results

We expect to find information on the facilitators and barriers to the implementation of digital interventions for people with dementia or their caregivers. We expect to provide information for politics and practice and to extract guidance for future research.

The search and preliminary analysis were carried out between May 5, 2023, and August 1, 2024. A total of 612 studies were identified across 3 databases. After the removal of duplicates, 400 articles remained. Title and abstract screening further reduced this number to 20 articles for full-text analysis. Ultimately, 5 studies met the inclusion criteria, focusing on the acceptability and barriers to technology use among individuals with dementia or their caregivers. Results were analyzed between August 2024 and October 2024, and the results are expected to be published later in 2025.

## Discussion

### Overview

As far as we know, this will be the first umbrella review addressing acceptance factors and barriers to the implementation of digital interventions in older people with dementia and their caregivers. Providing information on the facilitators and barriers to the implementation of digital interventions in people with dementia and their caregivers is extremely relevant from a clinical perspective [6,7,9,13,17,19,22,25]. This debate, present in the literature, encompasses several key issues: the effectiveness of digital interventions, the acceptability and usability of these technologies by older adults with dementia, and the various barriers to successful implementation, such as technological complexity and lack of digital literacy [12-14]. Additionally, there is concern about the potential for digital technologies to increase the burden on caregivers, who must learn and manage new technologies while providing care [12-14]. Discussions also focus on how digital technologies can improve QoL and health outcomes for people with dementia and their caregivers, including assessing clinical effectiveness and the impact on daily living and well-being [13,25]. Furthermore, the economic feasibility and broader social implications of implementing digital technologies in dementia care have been debated, including considerations of cost-effectiveness and potential health care cost reductions [6,7,25]. By systematically reviewing and synthesizing the available literature, this review seeks to provide a clearer understanding of these issues, thereby contributing to the theoretical debate and offering insights for future research and policy development.

Our umbrella review can significantly contribute in several ways, as noted in the following paragraphs.

It can identify barriers and facilitators. By synthesizing the results of various systematic reviews and meta-analyses, this review identifies common barriers and facilitators for implementing digital technologies in dementia care. This includes technical, financial, social, and psychological aspects that influence the accessibility and effectiveness of these interventions [12-14].

It can provide information for policies and practices. Insights obtained from this review can inform the formulation of policies and clinical practices, helping to shape guidelines that encourage the adoption of effective and sustainable technologies in dementia care [6,7,25].

Guidance for future research will be obtained. By highlighting gaps in the existing literature, this review can point to areas that need further investigation. This includes the need for studies exploring new technologies as well as studies examining diverse populations and different care contexts [12-14].

The review will also highlight research gaps in the field, pointing to associations and issues that have not been adequately explored. For example, there may be a lack of studies evaluating the impact of emerging technologies or considering the cultural and socioeconomic variability among different groups of patients and caregivers. Additionally, there may be a need for investigations into the long-term effects of using digital technologies in dementia care [12-14].

Overall, this umbrella review will contribute to the current debate on the value of a technological approach in dementia care. By providing a holistic view of the facilitators and barriers, this review can promote a more informed discussion on how to better integrate technology into geriatric care, potentially leading to better support for the autonomy and QoL of patients and their caregivers.

In summary, this review offers a unique opportunity to consolidate and critically evaluate the existing evidence on the acceptance and barriers to digital interventions in dementia care. Its findings can influence both clinical practice and future research, contributing to a better understanding and implementation of these technologies in a context that continues to grow in importance due to the aging global population.

### Limitations

To the best of our knowledge, this is the first general review that specifically addresses issues related to acceptability factors and barriers to the use of digital technology in people with dementia and caregivers. However, we expect to find difficulties: We expect to find systematic reviews with different protocols and different populations with different objectives. That will make the possibility of reaching final conclusions more difficult and, in certain cases, unobtainable.

## Abbreviations

AMSTAR: A Measurement Tool to Assess Systematic Reviews
CFIR: Consolidated Framework for Implementation Research
PICOS: population, intervention, comparison, outcomes, and study design
PRIOR: Preferred Reporting Items for Overviews of Review
PRISMA: Preferred Reporting Items for Systematic Reviews and Meta-Analyses
QoL: quality of life
WHO: World Health Organization

## Data Availability

The data sets generated or analyzed during this study are available from the corresponding author upon reasonable request.
